# Next-generation Sequencing Reveals Age-dependent Genetic Underpinnings in Lung adenocarcinoma

**DOI:** 10.7150/jca.65370

**Published:** 2022-03-06

**Authors:** Xiaonan Wu, Jun Zhao, Ling Yang, Xin Nie, Zheng Wang, Ping Zhang, Chao Li, Xueqing Hu, Min Tang, Yuting Yi, Xinhua Du, Xuefeng Xia, Yanfang Guan, Zicheng Yu, Wenguang Gu, Xiangming Quan, Lin Li, Hong Shi

**Affiliations:** 1 Department of Medical Oncology, Beijing Hospital, National Center of Gerontology; Institute of Geriatric Medicine, Chinese Academy of Medical Sciences, Beijing, P. R. China; 2 Department of Thoracic Medical Oncology, Peking University, School of Oncology, Beijing Cancer Hospital & Institute, Beijing, P. R. China; 3 Department of Gerontology, Beijing Hospital, National Center of Gerontology; Institute of Geriatric Medicine, Chinese Academy of Medical Sciences, Beijing, P. R. China; 4 Department of Pathology, Beijing Hospital, National Center of Gerontology; Institute of Geriatric Medicine, Chinese Academy of Medical Sciences, Beijing, P. R. China; 5 Geneplus-Beijing, Beijing, 102206, P. R. China; 6 Geneplus-Shenzhen Genomics Institute, Shenzhen 518000, China

**Keywords:** Lung adenocarcinoma, Age stratification, Next-generation sequencing, Tumor Mutation Burden.

## Abstract

**Background**: More than 40% of lung cancer patients are diagnosed at ages over 70. However, the genomic and clinical characteristics among them remain elusive. Here, we performed targeted capture sequence to characterize the mutational spectrum of Chinese lung adenocarcinoma (LUAD) patients across ages.

**Patients and Methods**: 2025 LUAD patients were divided into three groups: young (≤50 years old) (n=416, 20.54%), intermediate (51~69 years old) (n=1271, 62.77%), and aged (≥70 years old) (n=338, 16.69%). 1,021-gene panel and 59-gene panel were used for sequencing with tissue samples. Genetic alterations and tumor mutation burden (TMB) in LUAD patients were investigated.

**Results**: The frequency of mutations affecting 20 genes were significantly higher in aged group than in young group, and fourteen of them were not reported before, including involved in cell cycle/apoptosis signaling (*FAT1, FAT2*), DNA damage repair (*FANCA* and* FANCM*), chromatin histone modification (*KDM6A*), *RTK/RAS/PI3K* signaling (*FLT4* and *MTOR*), *NOTCH* signaling (*NOTCH1, NOTCH2* and* NOTCH4*) and other signaling pathway or cellular regulatory factor (*KEAP1, ASXL1, EPHB1* and *ABCB1*). Six previously reported mutated genes (*RBM10*, *KRAS*, *LRP1B*, *CDKN2A* and *KMT2C/D*) were also significantly more frequent in aged group. Among clinical actionable mutation sites, *KRAS* mutation was presented more common in aged group; both *MET* exon 14 skipping and *MET* amplification were significantly positively correlated with old age; the fusions of *ALK*, *ROS1, RET and ERBB2* exon 20 insertion were less frequent in aged group. Furthermore, a higher level of TMB was found in aged group compared with young group.

**Conclusion:** In this study, we revealed the differences of somatic genetic mutations and TMB between young and aged LUAD patients, which may provide directions of targeted therapy and advantages of immunotherapy for the elderly in the future.

## Introduction

The number of lung cancer cases in China has been increasing year by year, which become a major problem in public health 1. Lung adenocarcinoma (LUAD) is the most common histological subtype. Although targeted therapy and immunotherapy of LUAD have made rapid progress in the past two decades, there are still many problems that have not been resolved 2.

Immunotherapy and targeted therapy, especially targeted therapy, are associated with the abnormalities of some tumor-related gene in lung cancer. Previous studies have shown some differences in genetic changes in different age groups, but some research conclusions are not consistent, such as the *EGFR* mutations between young and aged remained controversial 3-8. Meanwhile, the continuous development of new targeted drugs such as *KRAS* targeted drugs requires us to focus on more genes 9. Recently, research on immune-related biomarkers is also widely implemented, such as TMB 10 and some tumor suppressor genes* TP53, STK11, CDKN2A, KEAP1, LRP1B* 11-14. Given that lung cancer is a high-incidence malignant tumor mainly in the elderly, it is crucial to explore the genetic profile and TMB status of the aged patients.

In this study, we analyzed 2025 LUAD patients from China and performed targeted capture sequence of their tumor samples to identify genetic alterations in patients with different age. We also evaluated TMB in LUAD patients, providing valuable genetic information for treatment of immune checkpoint blocker (ICB).

## Materials and Methods

### Patient and Sample Collection

We retrospectively analyzed 2025 patients with LUAD who underwent next-generation sequencing (NGS) testing between 2014 and 2019. The diagnosis of LUAD was confirmed by at least two independent pathologists. Inclusion criteria included: (i) untreated LUAD; (ii) tumor samples require more than 20% of tumor cells; (iii) pleural fluid cytology samples need to be made into wax blocks. Exclusion criteria included: (i) LUAD diagnosed only by cytological smear; (ii) mixed with other histological types besides adenocarcinoma. The age of 50 have been identified as a threshold of young age and the aged patients were defined as ≥70 years old at NSCLC diagnosis in previous studies 15, 16. The patients in our study were divided into three groups: the young group (≤ 50 years old) (416, 20.54%), the intermediate group (51~69 years old) (1271, 62.77%), and the aged group (≥ 70 years old) (338, 16.69%) (Table S1). The median ages of the three groups were 46, 61 and 75, respectively. Only tissue samples were included in this study, including 1733 formalin fixed, paraffin - embedded (FFPE) tissues and 292 frozen tumor tissues. Data of LUAD stage and other clinical factors were shown in Table S1. As for tumor matched control sample, 2 ml blood was collected from each patient. Written informed consent was obtained from each patient. This study was approved by the Institutional Review Board of Chinese Academy of Medical Sciences and conducted in accordance with the Declaration of Helsinki.

### Targeted-capture Sequencing

Targeted-capture sequencing was performed on Geneplus Seq-2000 platform as previously described 17-19. Briefly, ≥ 100ng of DNA was extracted from FFPE specimens or frozen tumor tissues. The purity and concentration of DNA were determined using Nanodrop 2000 spectrophotometer and Qubit 2.0 Fluorometer with Quant-IT dsDNA HS Assay Kit (Thermo Fisher Scientific), respectively. Library construction was carried out according to the protocols of KAPA Library Preparation Kit (Kapa Biosystems), and then capture hybridization was performed according to the manufacturer's protocol. DNA libraries sequencing was carried out utilizing Geneplus Seq-2000 (GenePlus, Beijing, China) with paired-end reads, with the support from a commercial vendor (Geneplus-Beijing, China).

Sequencing data were mapped to the reference human genome (hg19) and aligned using BWA version 0.5.9 (Broad Institute) after removal of terminal adaptor sequences and low-quality data. Tumor samples and matched control blood samples (white blood cells) were used to differentiate somatic mutations from germline polymorphisms. Single nucleotide variants were called using MuTect (version 1.1.4) 20. Small insertions and deletions were determined by GATK 21. Copy number variations (CNV) were detected by CONTRA (2.0.8) 22. An in-house algorithm was employed to identified split-read and discordant read-pair to identify gene fusion. All final candidate variants were manually verified with the integrative genomics viewer browser.

### Analysis of Somatic Mutations and TMB

Three different panels, 1021-gene panel V1 and V2, and 59-gene panel, were used to sequence 2025 samples. The details of these panels have been reported previously 17-19. The whole-coding regions and fusion-related introns of *EGFR, KRAS, ALK, ROS1, RET, BRAF, MET, ERBB2* and *TP53* were well covered by all three panels. Due to the difference among panels, we did not use all of the 2025 samples in the following analysis. In the mutational spectrum and signature analysis, we only used 932 samples sequenced with 1021-gene panel V2. Given that the 59-gene panel only covered a small size of coding regions, we utilized 1124 samples sequenced by 1021-gene panel V1 covering ~ 0.7M coding regions and 1021-gene panel V2 covering 1M coding regions in the TMB analysis.

The number of somatic coding nonsynonymous single nucleotide variants, and insertions and deletions mutations per megabase (Muts/Mb) of genome examined was defined as TMB. Mutational signatures were profiled using R package deconstruct Sigs v1.9.0 23. The average sequencing depth for 1021-gene panel V1, 1021-gene panel V2 and 59-gene panel sequenced tumor samples were 1247X, 1187X and 1636X accordingly, which were suitable for variant calling and quality assurance of variant reads.

### Statistical Analysis

The experimental data were presented as mean ± standard deviation. Chi-squared test, Fisher's exact test and Mann-Whitney test were used in statistical comparison of patients' clinicopathological characteristics, distribution of mutations in driver genes and TMB levels across three groups when appropriate. All tests were two-sided and considered statistically significant at *p*-values < 0.05. Binomial logistic regression analyses were carried out to correct for important covariates such as sex. For investigating whether or not age was associated with specific mutations occurred in LUAD patients, the variate with a *p*-value < 0.05, detected in unadjusted analyses, was further tested with adjusted analyses. A *p*-value < 0.05 was considered significant in both unadjusted and adjusted analyses. All statistical analyses and data visualization were performed on R package deconstruct Sigs v1.9.0, GraphPad Prism (7.01) or SPSS software (Version 23.0.0, IBM Corp, Armonk, NY).

## Results

### Genomic Profiling and Mutational Signatures among Different Age Groups

**Figure 1:**
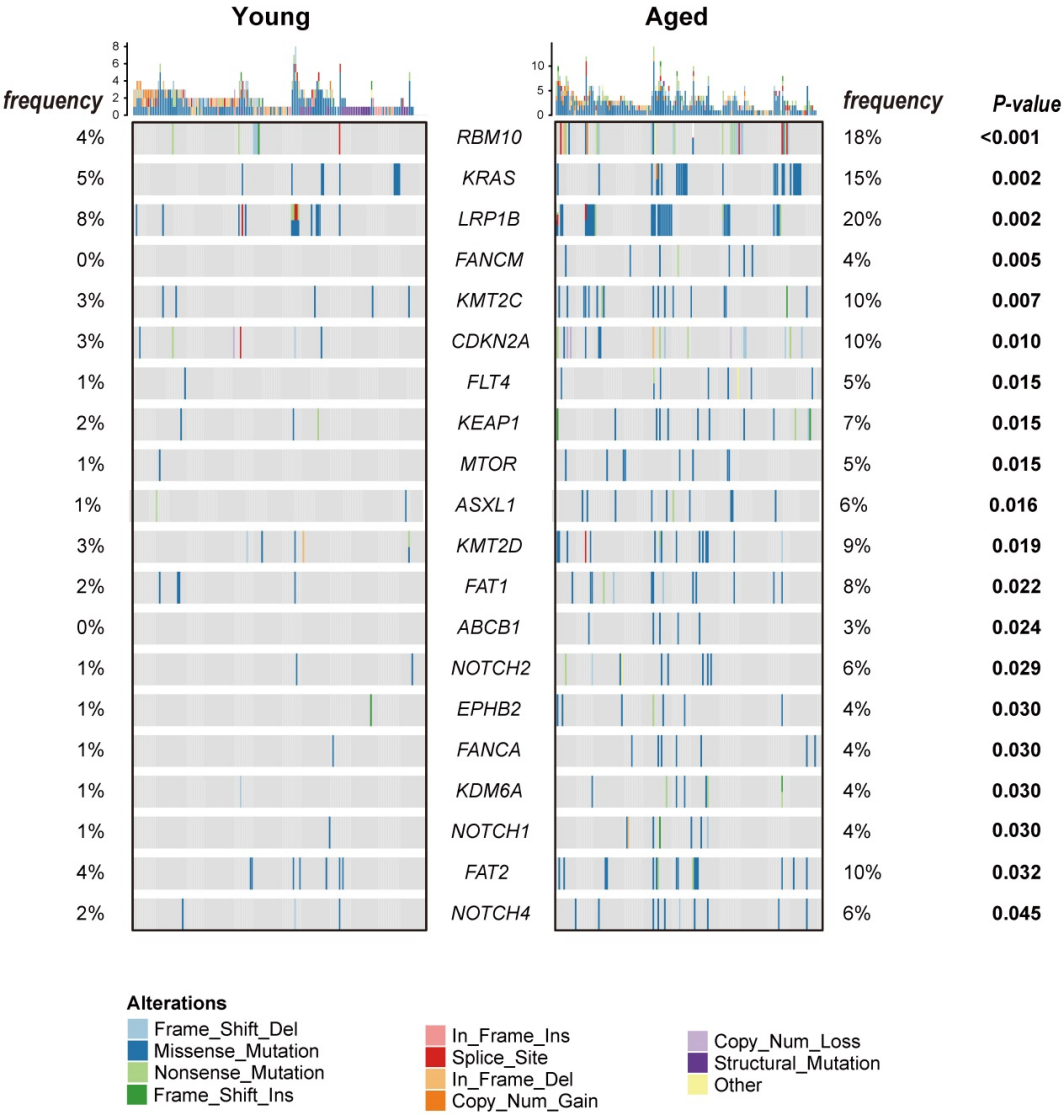
Heatmap of nonsynonymous somatic mutations with significantly different frequencies between the young and aged group. The types of nonsynonymous somatic mutations are shown in different colors. The *p*-value between the two groups was computed by Fisher's exact test and showed on the right.

**Figure 2:**
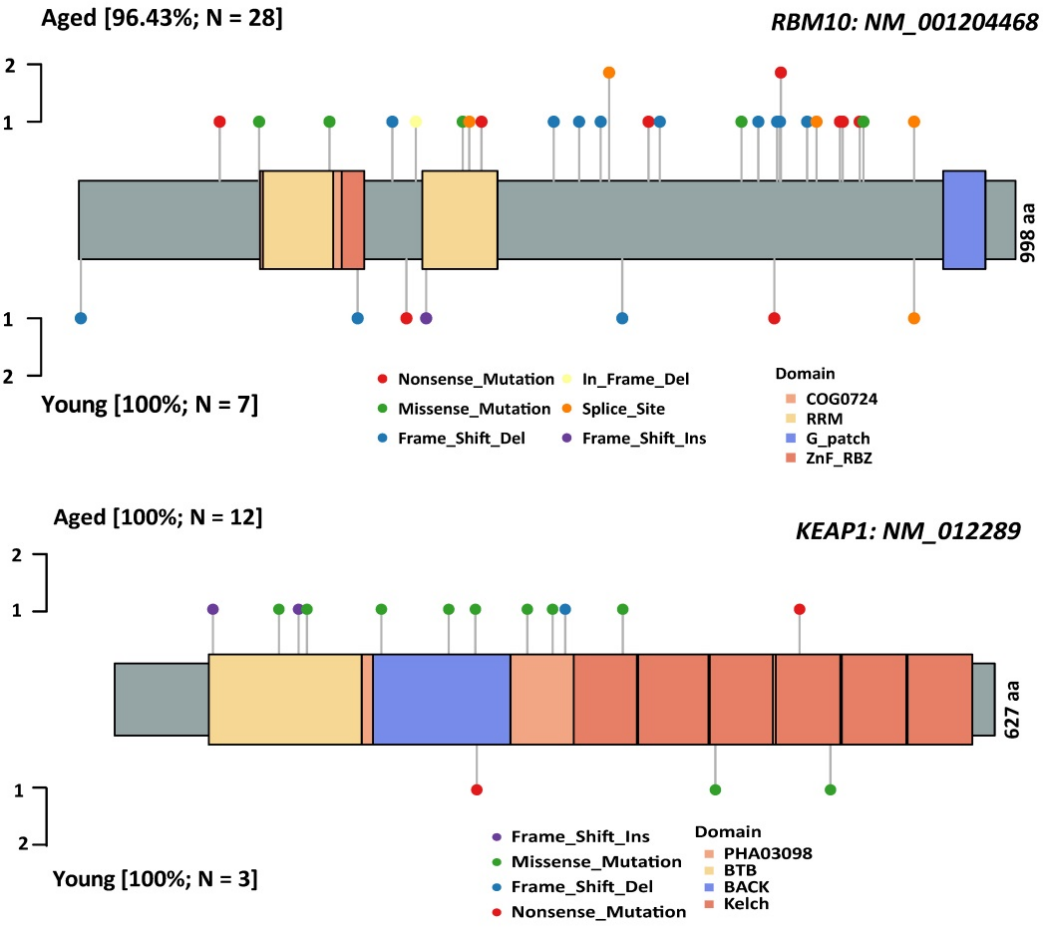
The comparison of spectrum of somatic mutations affecting *RBM10* and *KEAP1* between aged and young groups. A: *RBM10* gene; B: *KEAP1* gene. The types of somatic mutations and domains are shown in different colors.

Characterization of the genomic landscapes and mutational signatures of 932 LUAD tumor samples was performed using the data sequenced by the 1021 v2 panel. Nonsynonymous somatic mutations were identified in 97.96% (913/932) of patients (Figure S1). Twenty mutated genes were significantly more frequent in aged group than in young group, including 14 firstly-reported novel genes to the best of our knowledge (*FANCM*, *FLT4*, *KEAP1*, *MTOR*, *ASXL1*, *FAT1*, *ABCB1*,* EPHB2*, *FANCA*, *KDM6*, *FAT2,* and* NOTCH1/2/4*), and six previously reported genes (*RBM10* 24, *KRAS* 7,* LRP1B* 8*, CDKN2A* 25 and* KMT2C/D*^8^, ^26^) (Figure 1). Subsequently, Logistic regressions were used for investigating the association between age and genetic alteration after correction for gender. In result, there was significantly positive in 8 mutated genes (*RBM10, LRP1B, ASXL1, FAT1, MTOR, NOTCH2, CDKN2A, KEAP1*) (Table 1). Among these, *RBM10* enriched in aged group showed the most significant difference between groups. In addition, *KEAP1* mutations, potential marker of patients' resistance to immunotherapies, were also enriched in aged group. The ratios of truncated mutations to all types of mutation in *KEAP1* were both 1/3 in the two groups. Therefore, the incidence of truncated* KEAP1* mutations between aged group and young group was 0.0242 (4/165) and 0.005 (1/182) (*p* = 0.1952). The domains of *KEAP1* gene where mutational sites lied in were also different between two groups (Figure 2). The comparison of the structural domains where other mutations occurred between two groups was shown in Figure S2.

### The Association of Clinical Actionable Mutations with age

We further analyzed the relationship between age and alteration in eight clinical actionable genes in 2025 LUAD patients. Compared with the young group, patients in aged group displayed higher proportion of *KRAS* mutations (13.91% vs 5.77%, *p* = 0.0002), *MET* EX14 skipping (3.25% vs 0.72%, *p* = 0.0132) and *MET* amplification (1.78% vs 0.24%, *p* = 0.0495). Among the* KRAS* mutations, *KRAS* G12C and *KRAS* G12V were accounted for the majority part and still present more common in aged group(4.44% vs 1.68%, *p* = 0.0297). In contrast, *ALK* fusion (0.00% vs 13.70%, *p* < 0.0001), *RET* fusion (0.00% vs 2.88%, *p* = 0.0008) and *ERBB2* EX20 insertion (1.48% vs 5.77%, *p* = 0.0020) were found significantly less in aged group than in young group, while *ROS1* fusion tended to be more prevalent in the young group even without statistical difference.

Notably, no differences were found in *EGFR* mutations between groups, as well as *EGFR* exon21L858R and *EGFR* exon19del. *BRA*F V600E showed no difference either (Table 2). Higher proportion of *KRAS*-*TP53* co-mutation while less proportion of *EGFR-TP53* co-mutation were found in aged group than in young group (Figure S3A-B). *EGFR* exon 19 del and* TP53* were co-mutated more frequently in young group (30%) than in intermediate and older group, whereas no statistical difference was found in the co-mutation of *EGFR* exon 21L858R and TP53 between young and aged group (Figure S3 C-D), as well as *TP53* mutations and the specific mutations in *TP53* exon 5 and exon 8 (Figure S3 E-F).

### The Characteristics of TMB across ages

**Figure 3:**
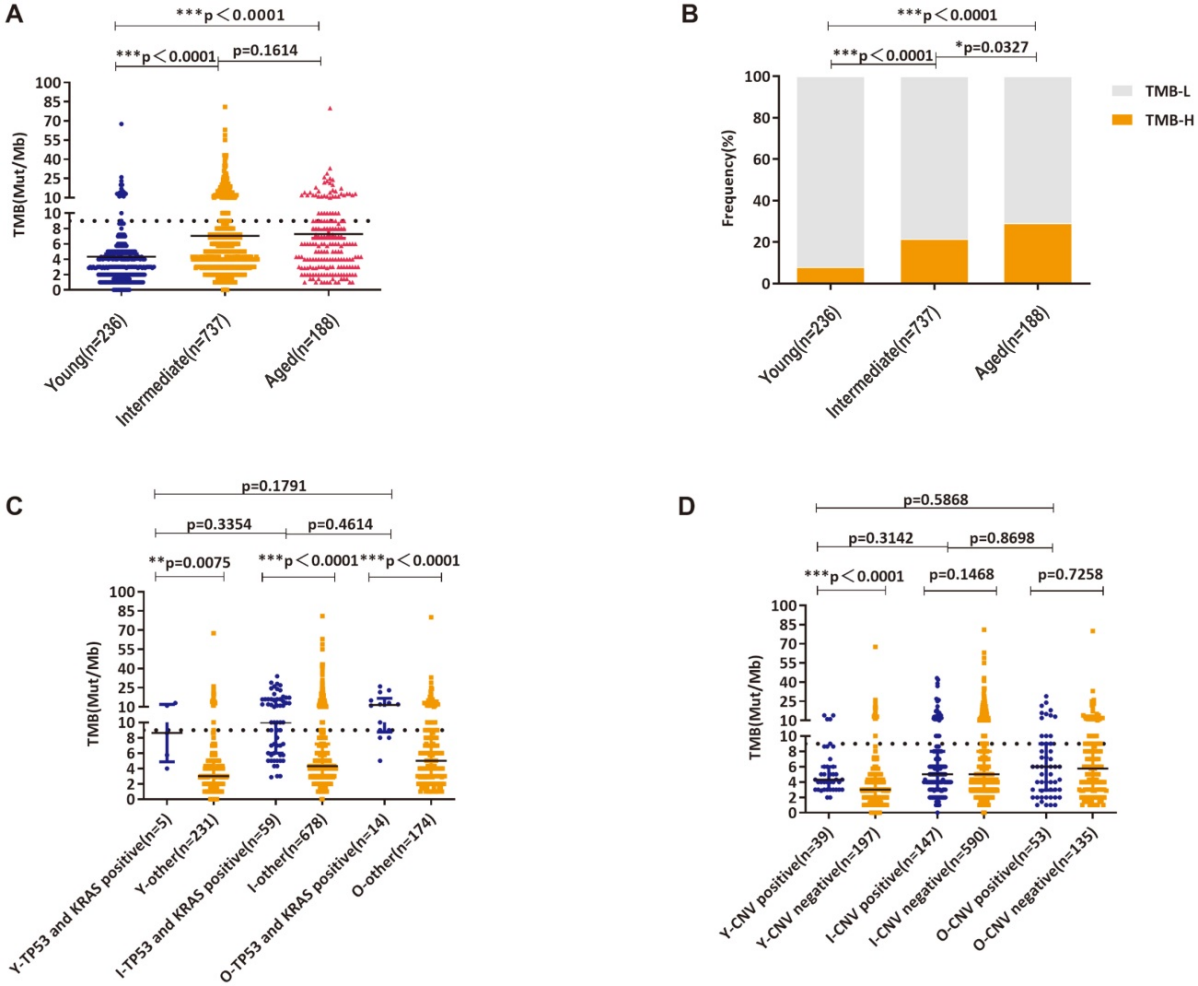
TMB distribution and High-level TMB in three age groups; A: Analysis of TMB distribution; B: Analysis of High-level TMB and Low-level TMB distributions; C: Analysis of TMB distributions in patients with concurrent *KRAS/TP53* mutations and wild-type *KRAS/TP53*; D: Analysis of TMB distributions in patients with altered copy numbers and wild-type copy numbers(Y—young, I—intermediate, O—old). *, ** and *** indicates *p* < 0.05, *p* < 0.01 and *p* < 0.001, respectively.

In this study, the median TMB in young, intermediate and aged group were 3 muts/Mb, 5 muts/Mb and 5.88 muts/Mb, respectively (Figure 3A). Using the upper quartile of the total population 9 muts/Mb as the threshold value, the proportion of TMB-H patients in each group was 8.05%, 21.57% and 29.26%, accordingly (Figure 3B). Similar to the previous findings, significantly higher TMB was observed in patients carrying* TP53* and *KRAS* mutations in all age groups (Figure 3C). In young group, patients with CNV displayed higher TMB than that of patients without CNV (Figure 3D).

### Mutational Signatures across Ages

The presence and relative contributions of single base substitution (SBS) signatures and doublet base substitution (DBS) signatures were determined in different age groups. The analysis revealed a signature of unknown origin in young group, characterized by transcriptional strand bias for T > C substitutions at 5'-ATN-3' context with more mutated A than T bases on the non-transcribed strands (termed SBS5) (Figure S4A). Moreover, signatures of DBS3 (associated with polymerase epsilon exonuclease domain mutations) and DBS7 (associated with defective DNA mismatch repair) were also observed in the young group (Figures S4B). Signatures SBS40 and DBS2 were discovered in the aged group (Figures S4A-B).

## Discussion

This work demonstrates so far the largest study comparing genetic characteristics between the aged and the young with newly diagnosed LUAD in China 8, 25, 27. In this study, we found that 14 genes which were not reported to date and six previously reported genes were more prevalently mutated in aged group. Notably, we observed that* CDKN2A KMT2C*, *KMT2D* mutations were enriched in aged group in our study, which is inconsistent with findings in previous literature 8, 25, 26 . Possible explanations of this discrepancy were the difference in sample sizes and thresholds to define young and aged. Of particular interest, in the aged group, mutated genes with extremely high prevalence exhibit great potential in developing matched therapies. These genes are roughly involved in cell cycle/apoptosis signaling pathways (*CDKN2A, FAT1, FAT2* and *RBM10*), DNA damage repair (*FANCA* and *FANCM*), chromatin histone modification (*KDM6A, KMT2C* and *KMT2D*), *RTK/RAS/PI3K* signaling pathways (*FLT4, KRAS* and* MTOR*) and NOTCH signaling pathway (*NOTCH1, NOTCH2* and *NOTCH4*) and other signaling pathway or cellular regulatory factor (*KEAP1, ASXL1, EPHB1* and *ABCB1*). The abnormality of these signaling pathways may be an important cause of LUAD tumorigenesis in the elderly. It is worth mentioning that *RBM10, KEAP1* and* LRP1B*, regarded as tumor suppressor genes, were more frequently mutated in aged group compared with young group, which may contribute to the high incidence of LUAD in the elderly.

Similar studies have analyzed the difference in distributions of actionable mutations across lung cancer patients with different ages 3, 25, 27. Within expectation, some conclusions from previous research were consistent with our findings in this study. Nevertheless, considering the modest sample sizes in previous research, this study provided more comprehensive information obtained from a substantially larger data set. As for LUAD patients with ages over 70 years, the opportunity to receive the treatment of targeted therapy was relatively lower. *RET, ALK* fusion and *ERBB2* exon 20 insertion occurred more frequently in young group, of particular note was the extremely low incidence of* RET* fusion (0/338). Frequencies of *EGFR* mutations were similar between young and aged groups, indicating that two groups tend to have the equal opportunity for *EGFR* targeted therapy. Although* EGFR 19del* mutations were a little more prevalent in young group than in aged group, the incidence of *TP53* mutations was also higher in young *EGFR* positive patients than in aged ones. As is known to all, EGFR-tyrosine kinase inhibitors (EGFR-TKIs) were more effective in patients with *EGFR19del* whereas *TP53* mutations reduce their sensitivity to EGFR-TKIs 30. Therefore, these two factors may balance each other.

*KRAS* was more frequently mutated in aged group 3, 28. *KRAS* G12C and G12V were relatively high in the types of *KRAS* mutations in LUAD. In the past, *KRAS* was a very difficult target for drug development. In May 2021, Sotorasib (AMG510), the first targeted drug for the *KRAS* G12C mutation, has been approved by FDA. Nowadays, *KRAS* has been one of the most popular targets and many *KRAS* inhibitors are under development 9. This may bring expectation to the aged LUAD patients with *KRAS* mutations.

We found that *MET* exon 14 skipping and *MET* amplification were significantly associated with cancer diagnosis at a late age. This was consistent with the large-scale study of Awad's which patients with *MET* 14 exon skipping were older (median 72.5 years old) than those with *EGFR* mutation (median 61 years old, P < 0.001) 29. *MET* mutation-targeting drugs are also rising stars and offering prospect hope to the aged people 30.

The development of novel immunotherapies is urgently needed by aged patients with lung cancer. In June 2020, FDA approved TMB-H as one of the indicators to guide immunotherapies, despite its inconsistent predictive value in ICB clinical trials 31. Problems still remains before translating TMB-guided immunotherapies into daily clinical application, such as the lack of a standardized calculating method and a commonly-accepted cutoff value to define high levels of TMB, and the comparability between different detection platforms et al. Despite this, the increase of TMB in aged patients presented in this study may raise the confidence of ICB application in aged patients and offer them more treatment opportunities 32, 33. Furthermore, larger gene sequencing panel could help to develop more markers to indicate patients that might benefit or not from immunotherapies, such as *KEAP1* mutations 34.

In this study we found differences on SBS and DBS signatures in different age groups. SBS5, DBS3 and DBS7 mutational burden were increased in young LUAD groups. SBS5 was clock-like in that the number of mutations in most cancers and normal cells correlates with the age of the individual 35. A recently study by Chinese scholars reported that endogenous mutational processes with the SBS5 mutational signatures were ubiquitous among normal tissues 36. The increasing of SBS5 mutational burden in young LUAD group might indicate premature accumulation of aging mechanisms in the body, thereby promoting tumor formation. Moreover, signatures of DBS3 (associated with polymerase epsilon exonuclease domain mutations) and DBS7 (associated with defective DNA mismatch repair) were also observed in the young group that predicting the relationship between defects in repair mechanisms and tumor formation. Signatures SBS40 and DBS2 were increased in the old group. Numbers of mutations attributed to SBS40 were correlated with patients' ages for some types of cancer. DBS2 that exhibits transcriptional strand bias with more GG>TT mutations than CC>AA on the untranscribed strands of genes, indicative of damage on guanine and repair by transcription coupled nucleotide excision repair, was associated with mutational processes of exposure to tobacco smoking as well as other endogenous and/or exogenous mutagens. Gene mutation signatures show the imprint in the genome induced by external factors such as smoking, internal repair mechanisms, aging and many other factors. These signatures manifested differently in different tissue types 35. These findings may suggest that the study of genetic markers can further reveal the pathogenesis of LUAD and explore the direction of research and treatment in different age groups.

In interpreting these findings, several limitations inherent in this study must be considered. The proportions of different age were imbalanced, which was tough to avoid in such a “real-world” study. In addition, due to the long and earlier period, the related clinical data were incomplete, such as tumor stage, were only found in 672 (33.2%) patients and lack of smoking status and associated treatment outcomes. All these limited the further analysis for the correlation between LUAD-related mutated gene and treatment efficacy.

## Conclusion

Our study deepened the understanding in genetic underpinnings of LUAD by demonstrating differential mutational features across ages. These disparities suggest that tumorigenesis mechanisms likely vary according to age. The aged patients have fewer benefit from existing target therapy based on *ALK, ROS1, RET* and *ERBB2.* However, based on the presence of more other pathway mutations and increased TMB, aged patients may benefit from immunotherapy and newly developed target such as *KRA*S and *MET.*

## Supplementary Material

Supplementary figures and table.

## Figures and Tables

**Table 1:** Logistic regression analyses investigating the association of gene alterations with age after the correction of sex.

The presence of mutations	Characteristics	Odds ratio	95% CI	*P*‐value
*RBM10*	*Model 1*			
	Age	5.410	2.285-12.810	0.0001*
	Sex	0.704	0.455-1.091	0.117
*LRP1B*	*Model 1*			
	Age	2.371	1.217-4.621	0.011*
	Sex	3.964	2.568-6.118	0.000
*ASXL1*	*Model 1*			
	Age	6.170	1.322-28.807	0.021*
	Sex	0.677	0.261-1.757	0.423
*FAT1*	*Model 1*			
	Age	3.487	1.108-10.969	0.0326*
	Sex	1.817	0.986-3.349	0.055
*MTOR*	*Model 1*			
	Age	9.728	1.2-78.881	0.0331*
	Sex	0.709	0.352-1.427	0.335
*NOTCH2*	*Model 1*			
	Age	4.751	1.005-22.464	0.0492*
	Sex	1.821	0.728-4.552	0.2
*CDKN2A*	*Model 1*			
	Age	2.865	1.001-8.200	0.0497*
	Sex	2.662	1.397-5.071	0.003
*KEAP1*	*Model 1*			
	Age	3.670	1.001-13.449	0.0497*
	Sex	10.269	3.658-28.823	0.000010

Model 1: *Model 1* adjusted for age and sex. * indicates statistical significance.

**Table 2:** The major driver gene mutation with specific site in different age groups in 2025 LUAD patients.

Driver gene mutation		Young (n=416)	Intermediate (n=1271)	Aged (n=338)	P-value (young vs old)
*EGFR*	total	52.64%	52.71%	51.18%	0.7143
	*EGFR* L858R	18.51%	25.18%	23.37%	0.1046
	*EGFR* EX19del	24.76%	21.09%	19.23%	0.0784
*ALK* fusion		13.70%	4.48%	1.18%	<0.0001*
*KRAS*		5.77%	13.22%	13.91%	0.0002*
*ERBB2* EX20 insertion		5.77%	2.36%	1.48%	0.0020*
*ROS1* fusion		2.64%	1.49%	1.48%	0.3167
*RET* fusion		2.88%	1.34%	0.00%	0.0008*
*BRAF* V600E		0.72%	0.79%	0.89%	1.0000
*MET* amplification		0.24%	1.18%	1.78%	0.0495*
*MET* EX14 skipping		0.72%	1.10%	3.25%	0.0132*

* indicates statistical significance.
