# Supplementary material for: Next-generation Sequencing Reveals Age-dependent Genetic Underpinnings in Lung adenocarcinoma
Source: J Cancer. 2022 Mar 6;13(5):1565–72. doi: 10.7150/jca.65370 (PMC8965110; doi:10.7150/jca.65370)
Supplement: Supplementary file 1 — Supplementary figures and table. [file jcav13p1565s1.pdf]

Table S1. Demographic and clinical characteristics of 2025 LUAD patients.

Patients	Total	Young (≤50 years)	Intermediate (51-69 years)	Aged (≥70 years)
	n=2025	n= 416	n=1271	n= 338
Age:Median(quartile,y)	61(53-67)	46(41-48)	61(56-65)	75(72-78)
Gender				
Male	1050	174	696	180
Female	975	242	575	158
Stage of LUAD				
I	100	25(6.01%)	65(5.11%)	10(2.96%)
II	43	6(1.44%)	28(2.20%)	9(2.66%)
III	106	25(6.01%)	61(4.80%)	20(5.92%)
IV	423	82(19.71%)	273(21.48%)	68(20.12%)
NA	1353	278(66.83%)	844(66.40%)	231(68.34%)
Sample type				
FFPE	1733	363	1079	291
frozen tumor tissue	292	53	192	47
Panel				
1021-gene panel v1	292	68	193	31
1021-gene panel v2	932	182	585	165
59-gene panel	801	166	493	142

Abbreviations: FFPE, formalin-fixed paraffin-embedded.

Figure S1. Nonsynonymous somatic mutations in genes with mutation frequency ≥ 2% in young group across Ages. The types of nonsynonymous somatic mutations are shown in different colors. The number of the total mutations in any given patient is plotted above the heatmap.

Figure S2. The comparison of spectrum of somatic mutations between aged and young groups. A: *ASXL1* gene, B: *CDKN2A* gene, C: *FAT1* gene, D: *LRP1B* gene, E: *MTOR* gene, F: *NOTCH2* gene. The types of somatic mutations and domains are shown in different colors.

Figure S3. Distributions of *TP53* and *TP53* co-mutations in three age groups. A: Analysis of the distribution of concurrent *KRAS/TP53* mutations including the mutations in *KRAS/TP53* and others (*EGFR/TP53*, *ALK/TP53* or no mutation); B: Analysis of distribution of concurrent *EGFR/TP53* mutations; C: Analysis of distribution of concurrent *EGFR* 19del/TP53 mutations; D: Analysis of distribution of concurrent *EGFR* L858R/TP53 mutations; E: Analysis of the distribution of *TP53*-exon5 mutations; F: Analysis of distribution of *TP53*-exon8 mutations. *, ** and *** indicates $p < 0.05$, $p < 0.01$ and $p < 0.001$, respectively.

Figure S4. Mutational Signatures across Ages. A: SBS; B: DBS. The presence and relative contributions of single base substitution (SBS) signatures and doublet base substitution (DBS) signatures were determined in different age groups.

Figure S1

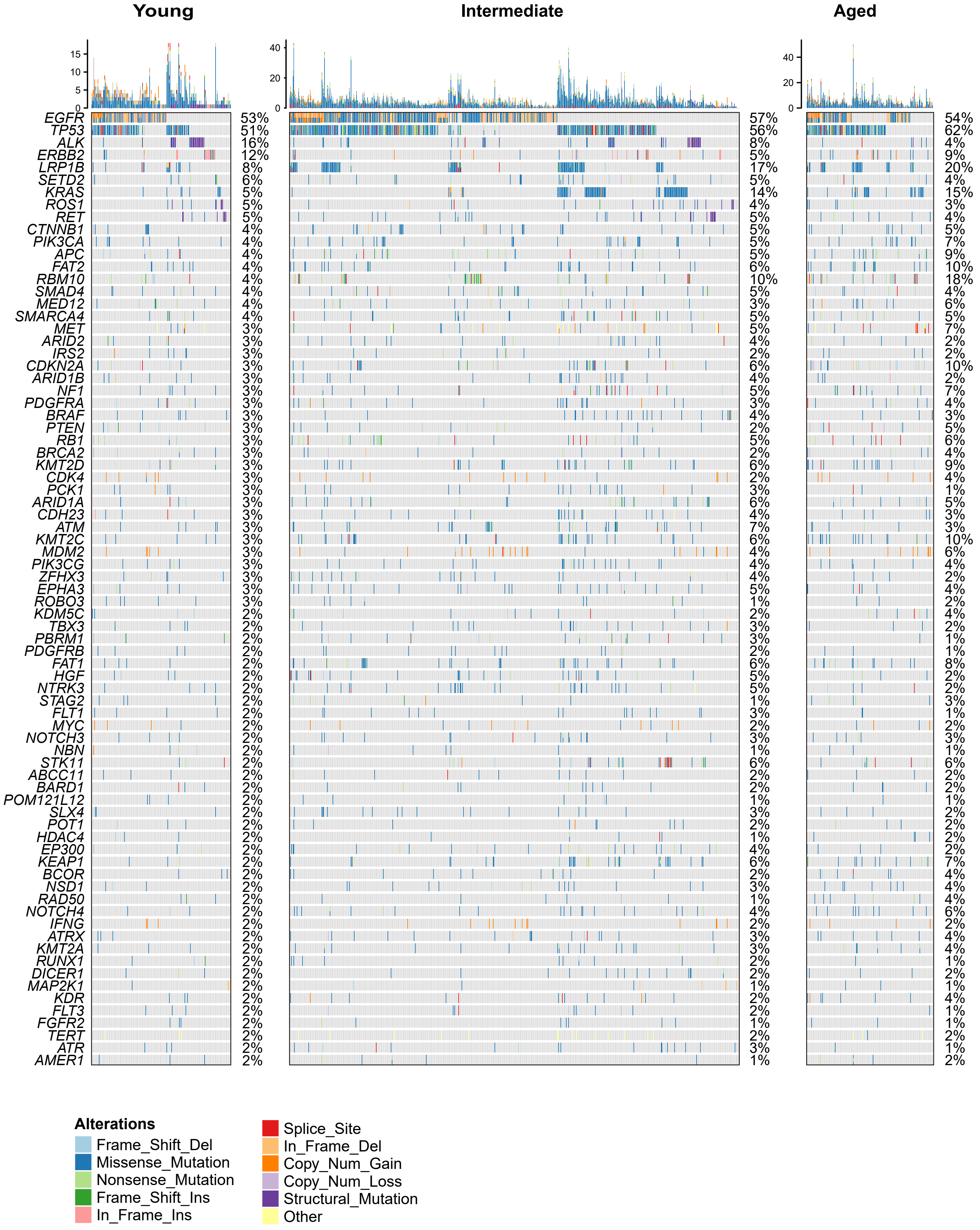
Young
Intermediate
Aged
15
10
5
0
40
20
0
40
20
0
EGFR 53% 57% 54%
TP53 51% 56% 62%
ALK 16% 8% 4%
ERBB2 12% 5% 9%
LRP1B 8% 17% 20%
SETD2 6% 5% 4%
KRAS 5% 14% 15%
ROS1 5% 4% 3%
RET 5% 5% 4%
CTNNB1 4% 5% 5%
PIK3CA 4% 5% 7%
APC 4% 5% 9%
FAT2 4% 6% 10%
RBM10 4% 10% 18%
SMAD4 4% 5% 4%
MED12 4% 3% 6%
SMARCA4 4% 5% 5%
MET 3% 5% 7%
ARID2 3% 4% 2%
IRS2 3% 2% 2%
CDKN2A 3% 6% 10%
ARID1B 3% 4% 2%
NF1 3% 5% 7%
PDGFRA 3% 3% 4%
BRAF 3% 4% 3%
PTEN 3% 2% 5%
RB1 3% 5% 6%
BRCA2 3% 2% 4%
KMT2D 3% 6% 9%
CDK4 3% 2% 4%
PCK1 3% 3% 1%
ARID1A 3% 6% 5%
CDH23 3% 4% 3%
ATM 3% 7% 3%
KMT2C 3% 6% 10%
MDM2 3% 4% 6%
PIK3CG 3% 4% 4%
ZFHX3 3% 4% 2%
EPHA3 3% 5% 4%
ROBO3 3% 1% 2%
KDM5C 2% 2% 4%
TBX3 2% 3% 2%
PBRM1 2% 3% 1%
PDGFRB 2% 2% 1%
FAT1 2% 6% 8%
HGF 2% 5% 2%
NTRK3 2% 5% 2%
STAG2 2% 1% 3%
FLT1 2% 3% 1%
MYC 2% 2% 2%
NOTCH3 2% 3% 3%
NBN 2% 1% 1%
STK11 2% 6% 6%
ABCC11 2% 2% 2%
BARD1 2% 2% 2%
POM121L12 2% 1% 1%
SLX4 2% 3% 2%
POT1 2% 2% 2%
HDAC4 2% 1% 2%
EP300 2% 4% 2%
KEAP1 2% 6% 7%
BCOR 2% 2% 4%
NSD1 2% 3% 4%
RAD50 2% 1% 4%
NOTCH4 2% 4% 6%
IFNG 2% 2% 2%
ATRX 2% 3% 4%
KMT2A 2% 3% 4%
RUNX1 2% 2% 1%
DICER1 2% 2% 2%
MAP2K1 2% 1% 1%
KDR 2% 2% 4%
FLT3 2% 2% 1%
FGFR2 2% 1% 1%
TERT 2% 2% 2%
ATR 2% 3% 1%
AMER1 2% 1% 2%
Alterations
Frame_Shift_Del
Missense_Mutation
Nonsense_Mutation
Frame_Shift_Ins
In_Frame_Ins
Splice_Site
In_Frame_Del
Copy_Num_Gain
Copy_Num_Loss
Structural_Mutation
Other

Figure S2

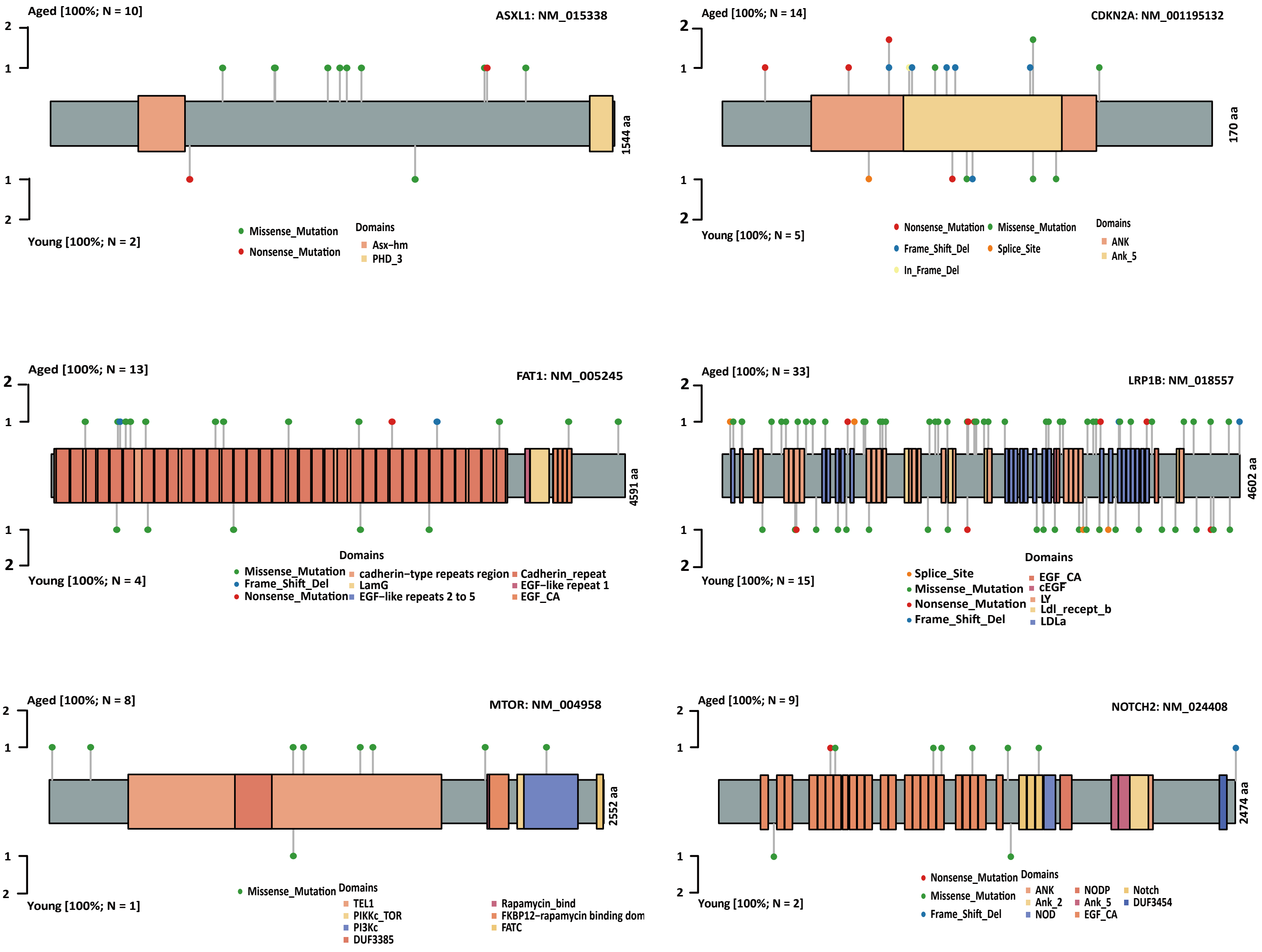

Aged [100%; N = 10]
ASXL1: NM_015338
1544 aa
Young [100%; N = 2]
Missense_Mutation
Nonsense_Mutation
Domains
Asx−hm
PHD_3
Aged [100%; N = 14]
CDKN2A: NM_001195132
170 aa
Young [100%; N = 5]
Nonsense_Mutation
Missense_Mutation
Frame_Shift_Del
Splice_Site
In_Frame_Del
Domains
ANK
Ank_5
Aged [100%; N = 13]
FAT1: NM_005245
4591 aa
Young [100%; N = 4]
Domains
Missense_Mutation
Frame_Shift_Del
Nonsense_Mutation
cadherin−type repeats region
LamG
EGF−like repeats 2 to 5
Cadherin_repeat
EGF−like repeat 1
EGF_CA
Aged [100%; N = 33]
LRP1B: NM_018557
4602 aa
Young [100%; N = 15]
Domains
Splice_Site
Missense_Mutation
Nonsense_Mutation
Frame_Shift_Del
EGF_CA
cEGF
LY
Ldl_recept_b
LDLa
Aged [100%; N = 8]
MTOR: NM_004958
2552 aa
Young [100%; N = 1]
Missense_Mutation
Domains
TEL1
PIKKc_TOR
PI3Kc
DUF3385
Rapamycin_bind
FKBP12−rapamycin binding dom
FATC
Aged [100%; N = 9]
NOTCH2: NM_024408
2474 aa
Young [100%; N = 2]
Nonsense_Mutation
Missense_Mutation
Frame_Shift_Del
Domains
ANK
Ank_2
NOD
NODP
Ank_5
EGF_CA
Notch
DUF3454

Figure S3

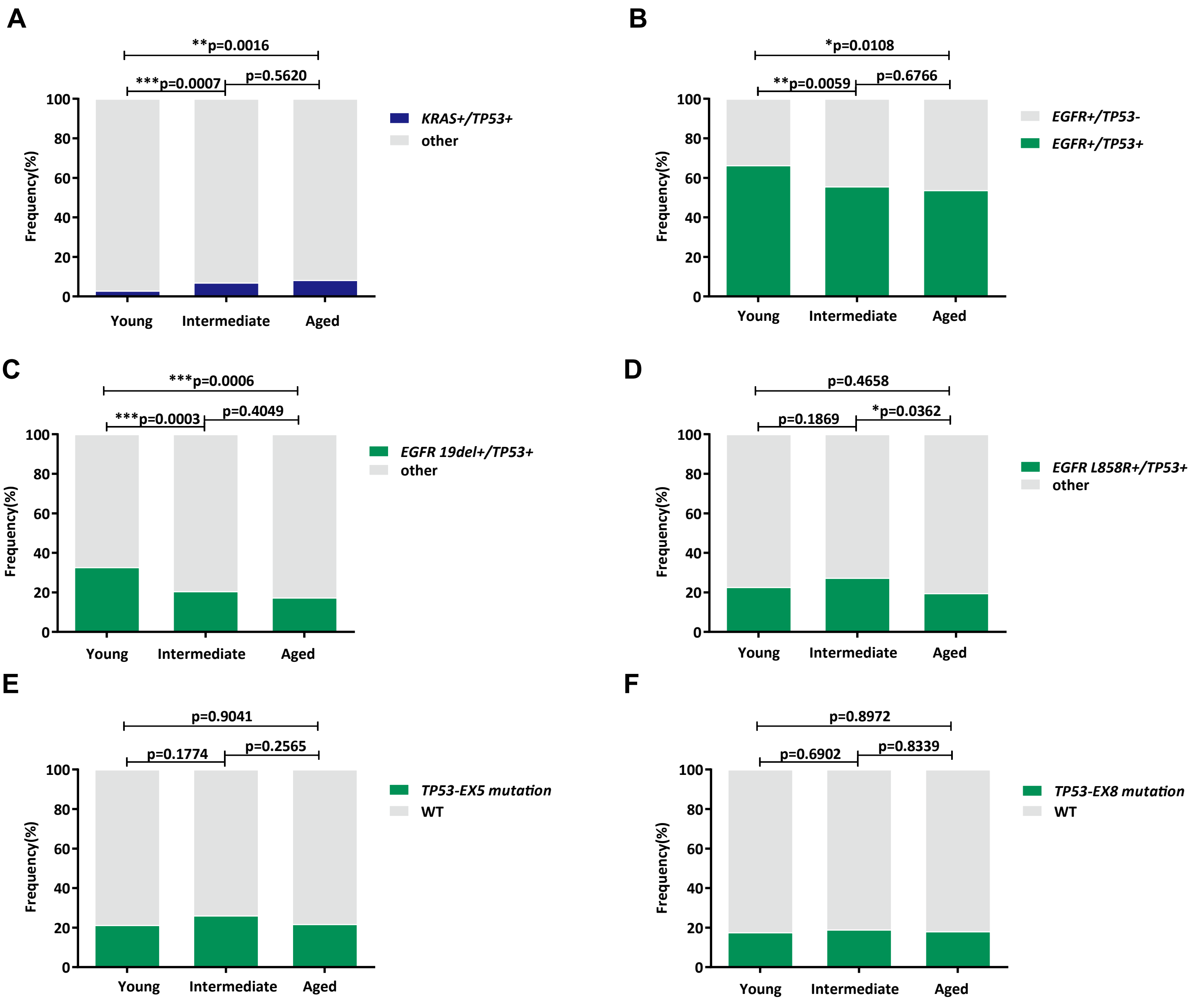

A
**p=0.0016
***p=0.0007
p=0.5620
Frequency(%)
100
80
60
40
20
0
Young
Intermediate
Aged
KRAS+/TP53+
other
B
*p=0.0108
**p=0.0059
p=0.6766
Frequency(%)
Young
Intermediate
Aged
EGFR+/TP53-
EGFR+/TP53+
C
***p=0.0006
***p=0.0003
p=0.4049
Frequency(%)
Young
Intermediate
Aged
EGFR 19del+/TP53+
other
D
p=0.4658
p=0.1869
*p=0.0362
Frequency(%)
Young
Intermediate
Aged
EGFR L858R+/TP53+
other
E
p=0.9041
p=0.1774
p=0.2565
Frequency(%)
Young
Intermediate
Aged
TP53-EX5 mutation
WT
F
p=0.8972
p=0.6902
p=0.8339
Frequency(%)
Young
Intermediate
Aged
TP53-EX8 mutation
WT

Figure S4

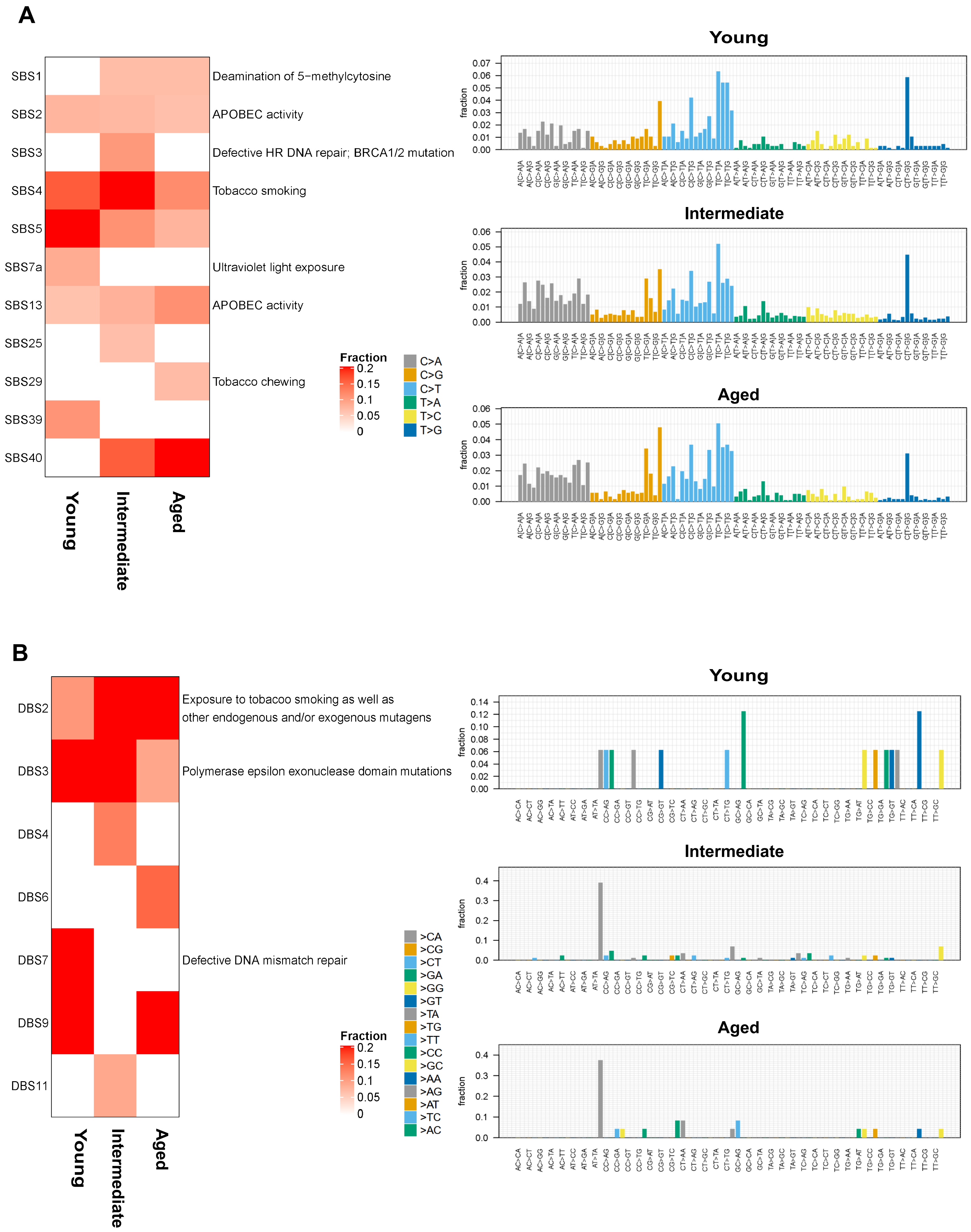
A
SBS1
SBS2
SBS3
SBS4
SBS5
SBS7a
SBS13
SBS25
SBS29
SBS39
SBS40
Deamination of 5−methylcytosine
APOBEC activity
Defective HR DNA repair; BRCA1/2 mutation
Tobacco smoking
Ultraviolet light exposure
APOBEC activity
Tobacco chewing
Young
Intermediate
Aged
Fraction
0.2
0.15
0.1
0.05
0
C>A
C>G
C>T
T>A
T>C
T>G
Young
Intermediate
Aged
fraction
B
DBS2
DBS3
DBS4
DBS6
DBS7
DBS9
DBS11
Exposure to tobacoo smoking as well as
other endogenous and/or exogenous mutagens
Polymerase epsilon exonuclease domain mutations
Defective DNA mismatch repair
Young
Intermediate
Aged
Fraction
0.2
0.15
0.1
0.05
0
>CA
>CG
>CT
>GA
>GG
>GT
>TA
>TG
>TT
>CC
>GC
>AA
>AG
>AT
>TC
>AC
Young
Intermediate
Aged
fraction
